# Three‐Dimensional Volume Ultrasound Assessment of Cesarean Scar Niche and Cervix in Pregnant Women: A Reproducibility Study

**DOI:** 10.1002/jum.16613

**Published:** 2024-11-07

**Authors:** Maria Ivan, Heba Mahdy, Amrita Banerjee, Amos Tetteh, Natalie Greenwold, Davide Casagrandi, Davor Jurkovic, Raffaele Napolitano, Anna L. David

**Affiliations:** ^1^ Fetal Medicine Unit University College London Hospital NHS Foundation Trust London UK; ^2^ Research Department of Maternal Fetal Medicine, Elizabeth Garett Anderson Institute for Women's Health, University College London London UK; ^3^ Department of Obstetrics and Gynaecology, Specialty Trainee Health Education England Thames Valley Oxford UK; ^4^ Department of Gynaecology University College London Hospital NHS Foundation Trust London UK; ^5^ Women's Health Theme, National Institute for Health Research, University College London Hospitals, Biomedical Research Centre London UK

**Keywords:** 3D ultrasound, cesarean birth, niche, reproducibility, scar, volume

## Abstract

**Objective:**

To assess the reproducibility of standardized 3‐dimensional (3D) ultrasound volume analysis of the dimensions and the position of cesarean birth (CB) scar niche relative to the cervix in pregnant women.

**Methods:**

This prospective single‐center study in women with 1 previous CB ≥8 cm cervical dilatation acquired ultrasound volumes between 11 and 24 weeks' gestation in a mid‐sagittal plane. Two experienced operators processed the volumes using virtual organ computer‐aided analysis. A CB scar niche was defined as an indentation at the scar site of ≥2 mm in depth. Niche and cervix volumes were calculated using manual contouring. Agreement for categorical variables was expressed using intraclass correlation coefficient (ICC). The Bland–Altman method was used to assess numerical variable reproducibility.

**Results:**

To achieve the desired statistical power, 52 participants were included. The intraobserver agreement on niche classification relative to the internal os was 100%, with an interobserver kappa coefficient of 0.98 (95% confidence interval [CI] 0.97–0.99, *P* < .05).

The intraobserver ICC for niche volume was 0.94 (95% CI 0.90–0.96; *P* < .001), with a mean difference of −15.32 mm^3^ (±109.32). The interobserver ICC was 0.78 (95% CI 0.62–0.87; *P* < .001), with a mean difference of −21.57 mm^3^ (±202.01). The ICC for niche/cervix volume ratio were 0.94 (95% CI 0.90–0.96; *P* < .001) and 0.79 (95% CI 0.63–0.87; *P* < .001) for intra‐ and interobserver reproducibility, respectively.

**Conclusions:**

This study demonstrates that 3D CB scar sonographic features are highly reproducible in pregnant women with a history of advanced labor CB. The validated protocol can guide future research on the association with subsequent adverse pregnancy outcomes.

## Abstract 2

Access the CME test here and search by article title.

**Abbreviations**
2D: two‐dimensional
3D: three‐dimensional
CB: cesarean birth
CI: confidence interval
ICC: intraclass correlation coefficient
LM: late miscarriage
sPTB: spontaneous preterm birth
US: ultrasound
VOCAL: virtual organ computer‐aided analysis

Cesarean birth (CB) rates are increasing worldwide.^1^ Advanced labor CB is linked to higher maternal and neonatal morbidity,^2^ as well as increased risk of late miscarriage (LM) or spontaneous preterm birth (sPTB) in future pregnancies, compared to elective or early labor CB.^3^, ^4^, ^5^, ^6^, ^7^

A CB scar niche, defined as an indentation at the site of the uterine scar with a depth measuring ≥2 mm on transvaginal ultrasound (US)^8^ is a long‐term complication of CB, describing an abnormally developed scar. Histological findings suggest that cesarean scars may heal poorly due to disruption in mucus formation, with studies documenting the presence of endocervical mucosa and glands at scar sites, particularly in cases with niche formation compared to those without defects.^9^, ^10^, ^11^, ^12^ Increasingly CB scar niche is being identified as a cause of gynecological symptoms (abnormal uterine bleeding, dysmenorrhea, and subfertility) and associated with major obstetric complications (placental accreta spectrum, cesarean scar pregnancy, and uterine rupture).^13^ Scar niche prevalence can reach 84%, but varies according to the assessment method, definition criteria and characteristics of the population studied.^14^ The position of the hysterotomy incision and the stage of labor at the time of CB impact on the characteristics of the scar niche.^15^

The relationship between 3‐dimensional (3D) US characteristics of poorly healed CB scars and subsequent adverse pregnancy outcomes has yet to be explored. Several studies focused on 2‐dimensional (2D) US assessment of CB scar, niche characteristics, and position relative to the internal os in pregnancy.^16^, ^17^, ^18^ Cervical volume can be reliably calculated from 2D images using a geometric formula or by 3D sonography.^19^, ^20^, ^21^ Previous studies on cervical volume found it was less useful than 2D US of cervical length in identifying women at risk of sPTB or in predicting vaginal birth after induction of labor.^22^, ^23^

Before scrutinizing the impact of 3D CB scar morphology on LM/sPTB and other adverse pregnancy outcomes, it is fundamental to define reproducible methods for sonographic assessment of the CB scar and its relation to the cervix in pregnancy. We prospectively investigated the feasibility and reproducibility of a standardized 3D US protocol for assessing the characteristics of the CB scar niche in pregnant women with a previous advanced labor CB.

## Materials and Methods

This was a prospective cohort study conducted at University College London Hospital, UK between December 2019 and May 2022. Singleton pregnant women with a history of 1 previous term (≥37 weeks) emergency CB, in late first stage or second stage of labor (≥8 cm cervical dilatation) were included. The US scan assessments were undertaken in a specialist antenatal preterm birth surveillance clinic and interventions (vaginal progesterone and/or cervical cerclage) were offered to all pregnant women at increased risk of sPTB or LM because of a shortening cervical length.

**Figure 1:**
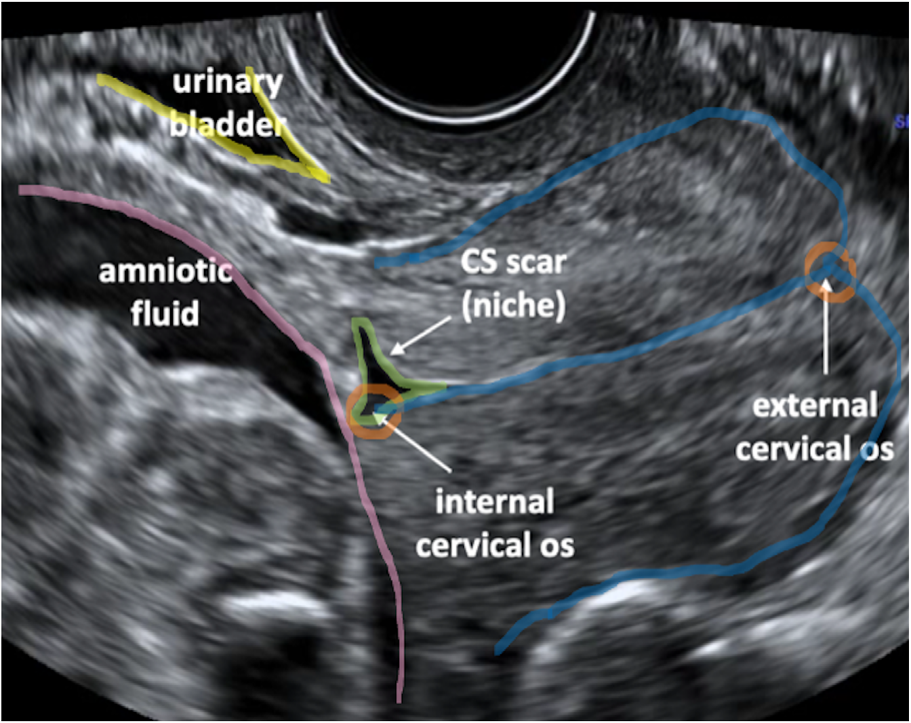
Transvaginal ultrasound image of the cervix and lower uterine segment highlighting the region of interest for 3D volume acquisition.

Transvaginal US imaging of the cervix and the lower uterine segment was performed on Voluson E8/E10 Expert Ultrasound Systems (GE Healthcare, Austria) using 5–9 MHz endovaginal 3D probes by 4 fetal medicine clinician operators (MI, AB, RN, and ALD). Three‐dimensional volumes were acquired during the second trimester of pregnancy, between 11 and 24 weeks of gestation, using a standardized protocol. The participants were examined in the presence of a chaperone and with consent, in supine gynecological position, post micturition. The region of interest for 3D volume acquisition included the longitudinal view of the entire cervix and lower uterine segment, as well as 3 key anatomical landmarks: the internal cervical os, cervical canal and external cervical os (Figure 1). The location of the internal cervical os was assessed by visualizing the whole cervical canal and using color Doppler mapping of the uterine arteries bilaterally in the paracervical region, as previously demonstrated by Banerjee et al.^16^ The cervix and lower uterine segment were magnified until they occupied 50%–75% of the screen. The automatic volume capture was achieved using a 120° acquisition angle and high image resolution option was activated. The acquisition time for each volume was 4–5 seconds during which time the participants were asked to remain still, which resulted in minimal motion artefacts. The US images and 3D volumes obtained were saved and stored securely, in accordance with UK General Data Protection Regulation (GDPR) policies.

Three‐dimensional sonographic volume processing was done offline using virtual organ computer‐aided analysis (VOCAL) imaging function of the 4D View software (GE Healthcare) on Windows 10 platform by 2 operators (MI and HM). Both examiners acquired specific dedicated skills as part of a comprehensive training program in obstetric US and practiced 3D niche and cervix volume analysis with a local fetal medicine expert in 3D VOCAL analysis (RN), prior to start of the study.^24^

**Figure 2:**
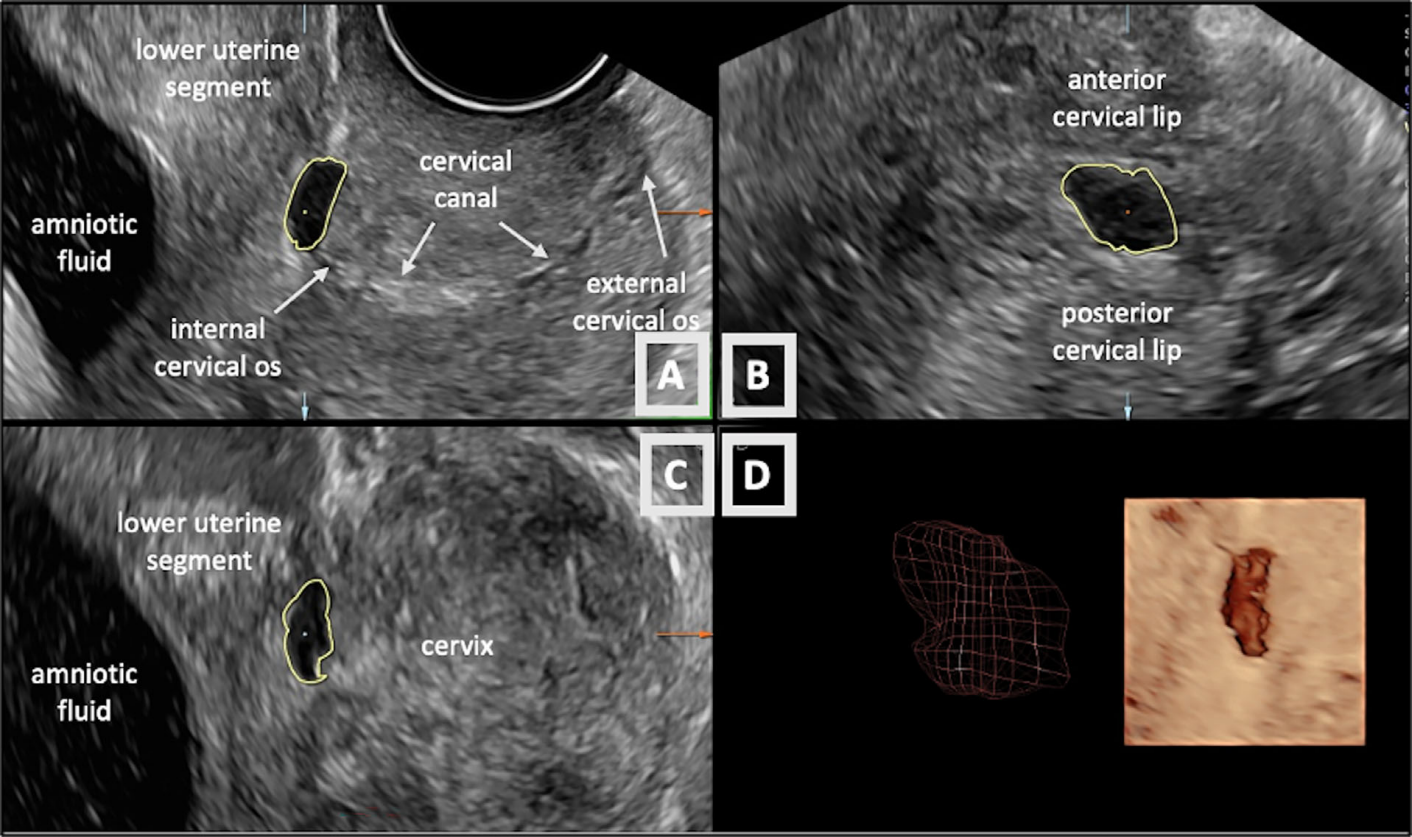
Manipulation of 3‐dimensional ultrasound volume of the lower uterine segment and cervix in sagittal (**A**), transverse (**B**), and coronal (**C**) planes using VOCAL. Three‐dimensional geometric reconstructions of a cesarean scar niche (**D**) measuring 209 mm^3^.

In the 3D volumes, the CB scar niche was defined as an “indentation at the level of the CB scar with a depth measuring ≥2 mm.”^8^ Each volume was displayed in 3 reference planes: sagittal (A), transverse (B), and coronal (C). By manipulating the image in the sagittal plane (A) along the *x*‐, *y*‐, and *z*‐axis and using “parallel shift” function (front to back) the niche was identified at its widest length and/or depth. Magnification and contrast functions were used in order to optimize niche visualization. The location of the internal os was identified dynamically in the sagittal plane, by reprocessing the volumetric data into slices using the “parallel shift” function of the software. A combination of methods was used to identify the level of the internal os: ending of the thin line of cervical mucosa, the continuation of the posterior vaginal fornix and the acute change in the uterine‐cervical axis (Figure 2).

**Figure 3:**
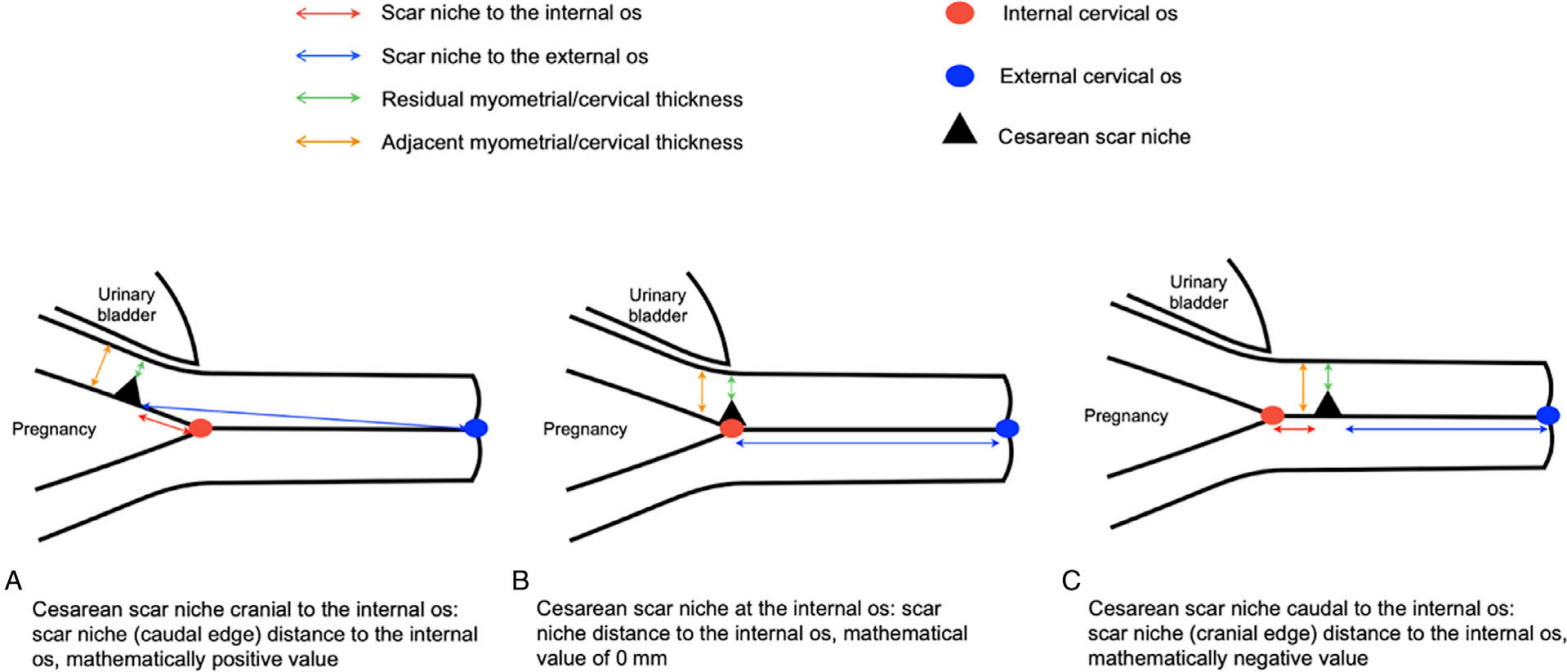
Schematic representation of cesarean scar niche measurements according to scar position relative to the internal cervical os.

The distance between the niche and the internal cervical os was determined by placing the calipers at the niche margin closest to the internal os and at the internal os itself. For niches positioned cranial to the internal os, the most caudal edge of the niche was used; while for niches caudal to the internal os, the most cranial edge of the niche was used. The caudal edge of the niche was used to determine the niche distance to the external cervical os. Niches were divided into 3 groups: cranial to, at the level of or caudal to the internal os. The following measurements were recorded using 2 calipers distance measurement function of the software in sagittal plane (A): shortest niche distance to internal and external cervical os, largest niche length, largest niche depth, shortest residual myometrial/cervical tissue thickness, and adjacent myometrial/cervical tissue thickness. Residual myometrial/cervical thickness was measured at its thinnest point, extending perpendicularly from the edge of the uterine niche to the uterine serosa and excluding any areas of fibrosis. Adjacent myometrial/cervical thickness was measured 5 mm cranially from the location of the residual myometrial/cervical thickness measurement (Figure 3). The image was then similarly manipulated in the transverse plane (B) to record the largest niche width.

**Figure 4:**
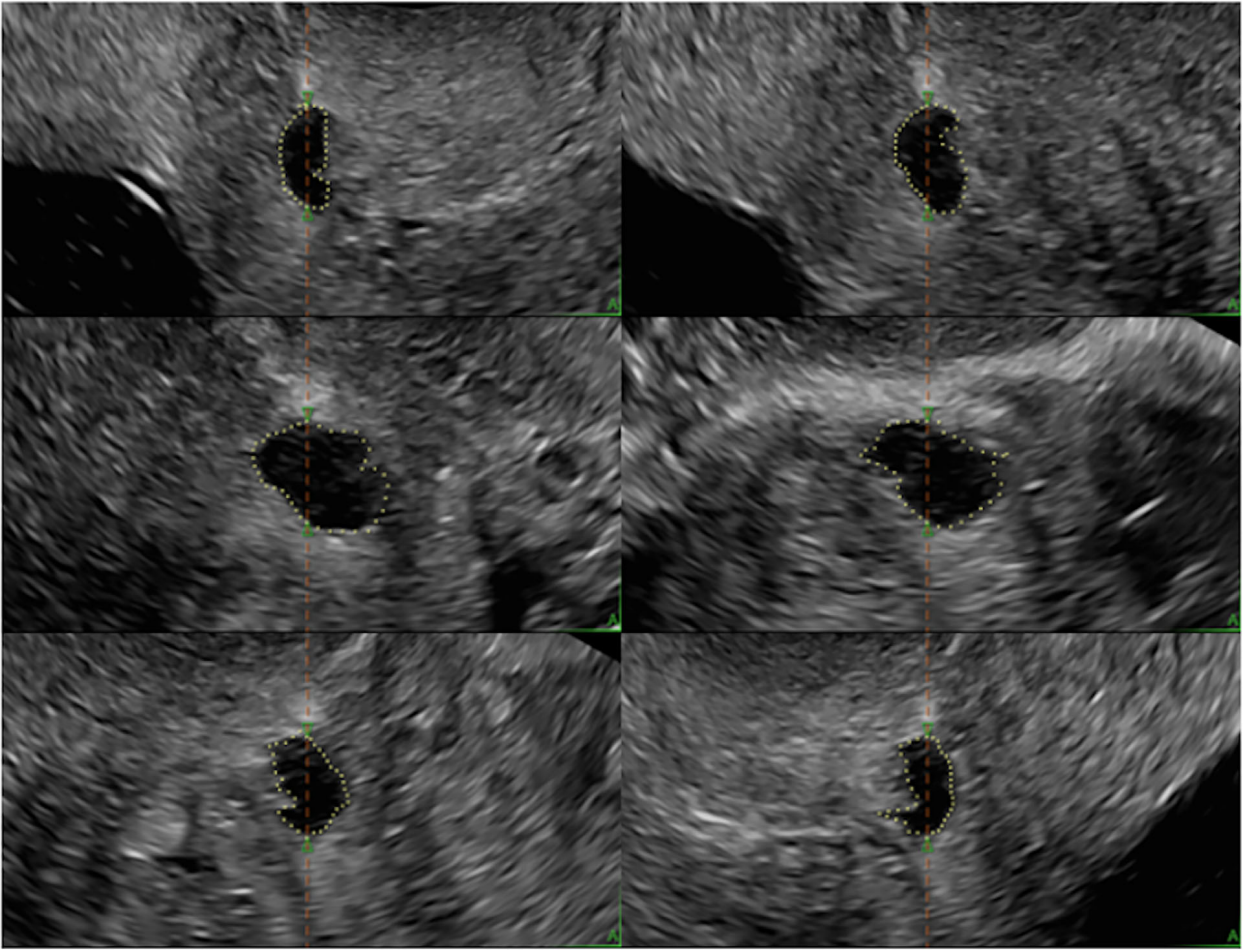
Three‐dimensional ultrasound volume of the lower uterine segment and cervix illustrating the manual contouring of a cesarean section scar niche technique in 6 reference planes at 30° angles from each other, starting in the mid‐sagittal plane (top left corner).

VOCAL mode was activated, and niche volume (mm^3^) was obtained using plane A as a reference plane. The reference dot was placed in the center of the niche in the mid‐sagittal plane and manual contouring of the niche was performed using 30° angle rotational steps (Figure 4). If a niche had multiple branches, manual contouring was done in a similar fashion and the total volume of the niche was recorded.

The optimal view of the cervix was identified by manipulating the image in the mid‐sagittal plane (A), ensuring that the anatomical landmarks described above were visualized. Cervical length was measured using calipers distance measurement function (straight line). Cervical volume was obtained using plane A as a reference plane. The image was rotated around the *z*‐axis in order to align the cervix in the horizontal plane and the reference dot was placed in the cervical canal, halfway between internal and external os. The cervical volume (mm^3^) was achieved using manual contouring of the cervix and 30° angle rotational steps. Niche ratio was calculated as a percentage of the niche volume to the cervical volume: Niche:cervix ratio = Niche volume/Cervix volume × 100.

The 3D sonographic volumes were analyzed in a random order by operator MI twice (4–8 weeks apart) for intraobserver reproducibility and by operator HM once for interobserver reproducibility. Operators were blinded to all previously recorded measurements. The intraobserver reproducibility was assessed based on first and second round of measurements taken by operator MI and the interobserver variability was calculated using operator MI's first measurements and operator HM's measurements. The mean of all 3 measurements was used to generate the distribution charts for niche measurements.

Statistical analysis was performed using IBM SPSS Statistics version 27. Level of agreement for categorical variables (niche identification and position in relation to the internal os) was expressed using intraclass correlation coefficient (ICC). ICC were calculated using a 2‐way mixed‐effects model, with fixed operators and random subjects from study cohort. We assessed ICC for absolute agreement to ensure exact measurement consistency between operators. A single‐measurement ICC was used to evaluate the reliability of assessments. Percentage of measurements within a predefined cut‐off were also analyzed as categorical variable. Bland–Altman method^25^ was employed to assess the reproducibility of numerical variables, using one‐sample *t* tests with a significance level of 0.05 to calculate the mean differences between individual measurements. Data were tested for normality using Shapiro–Wilk distribution test. A power analysis accounting for data from previous studies^16^, ^26^, ^27^, ^28^, ^29^, ^30^ estimated that an examination of 52 volumes would be necessary to identify significant variances among operators.

## Results

**Figure 5:**
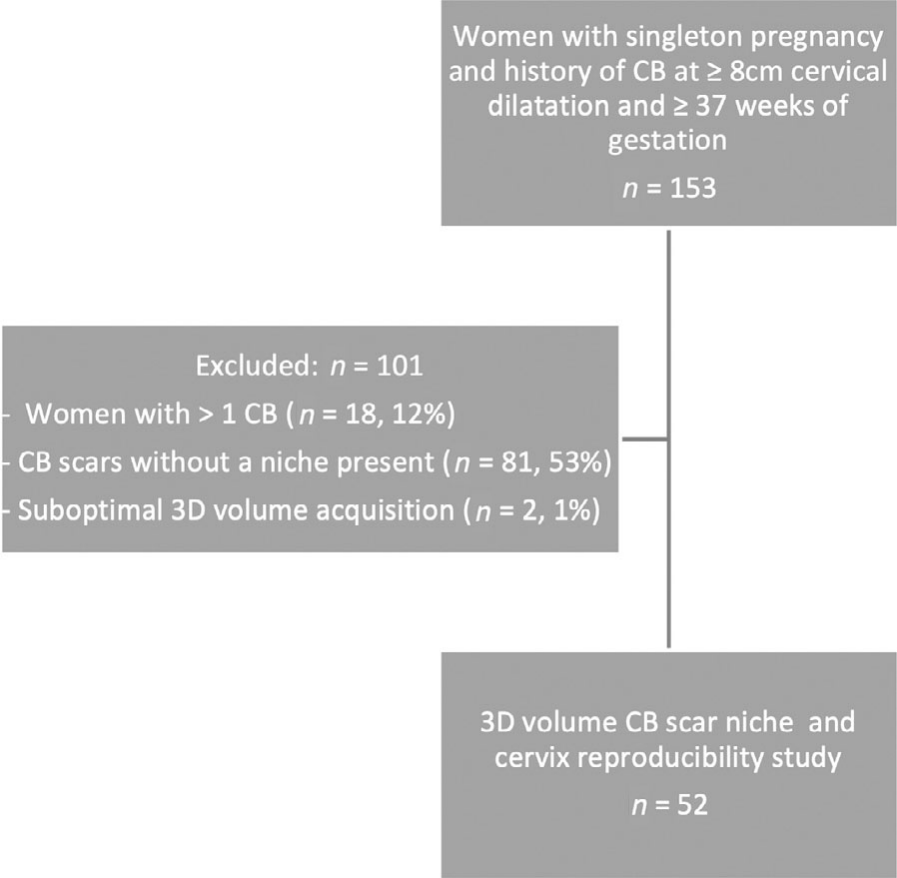
Flow diagram of the study population. CB, cesarean birth; 3D, 3‐dimensional.

A total number of 153 women who had a previous emergency CB at term in labor at ≥8 cm cervical dilatation had 3D sonographic volumes of the cervix and lower uterine segment acquired during the first and/or second trimesters of the subsequent pregnancy. Of these, we excluded 101 women from analysis, due to 1) having more than 1 CB (n = 18, 12%), 2) failure to identify a niche (n = 81, 53%), and 3) a suboptimal 3D volume acquisition (n = 2, 1%). This left 52 participants and their corresponding 3D transvaginal (TV) US volumes in the reproducibility study (Figure 5).

The median gestational age at the time of volume acquisition was 15 weeks' gestation and the median interval between the index CB and 3D US examination was 3 completed years (Table 1).

**Table 1:** Demographic and Obstetric Characteristics of the Study Population

Characteristic	Cases, n = 52 (%)	Median (±SD)
Age (years)		35 (±4.6)
18–34	25 (48.0%)	
35–40	20 (38.5%)	
≥40	7 (13.5%)	
Ethnicity		
White	32 (61.5%)	
Black	6 (11.5%)	
Southeast Asian	7 (13.5%)	
East Asian	2 (3.8%)	
Middle Eastern	4 (7.7%)	
Mixed other	1 (1.9%)	
BMI (kg/m^2^)		23.3 (±3.8)
≤18.5	1 (1.9%)	
18.6–24.9	33 (63.4%)	
25.0–29.9	13 (25.0%)	
≥30	5 (9.6%)	
Smoking status		
Nonsmoker	51 (98.1%)	
Current smoker	1 (1.9%)	
Method of conception		
Spontaneous	45 (86.5%)	
In vitro fertilisation	7 (13.5%)	
GA at CB (weeks)		40 (±1.6)
37–38	9 (17.3%)	
39–40	26 (50.0%)	
≥41	17 (32.7%)	
Interpregnancy interval (years)		3 (±2.9)
<1	2 (3.8%)	
1–5	35 (67.3%)	
≥ 5	15 (28.8%)	
GA at US examination (weeks)		15 (±3.1)
≤12	1 (1.9%)	
13–15	25 (48%)	
16–20	17 (32.7%)	
21–24	9 (17.3%)	

SD, standard deviation; BMI, body mass index; GA, gestational age; CB, cesarean birth; US, ultrasound.

**Figure 6:**
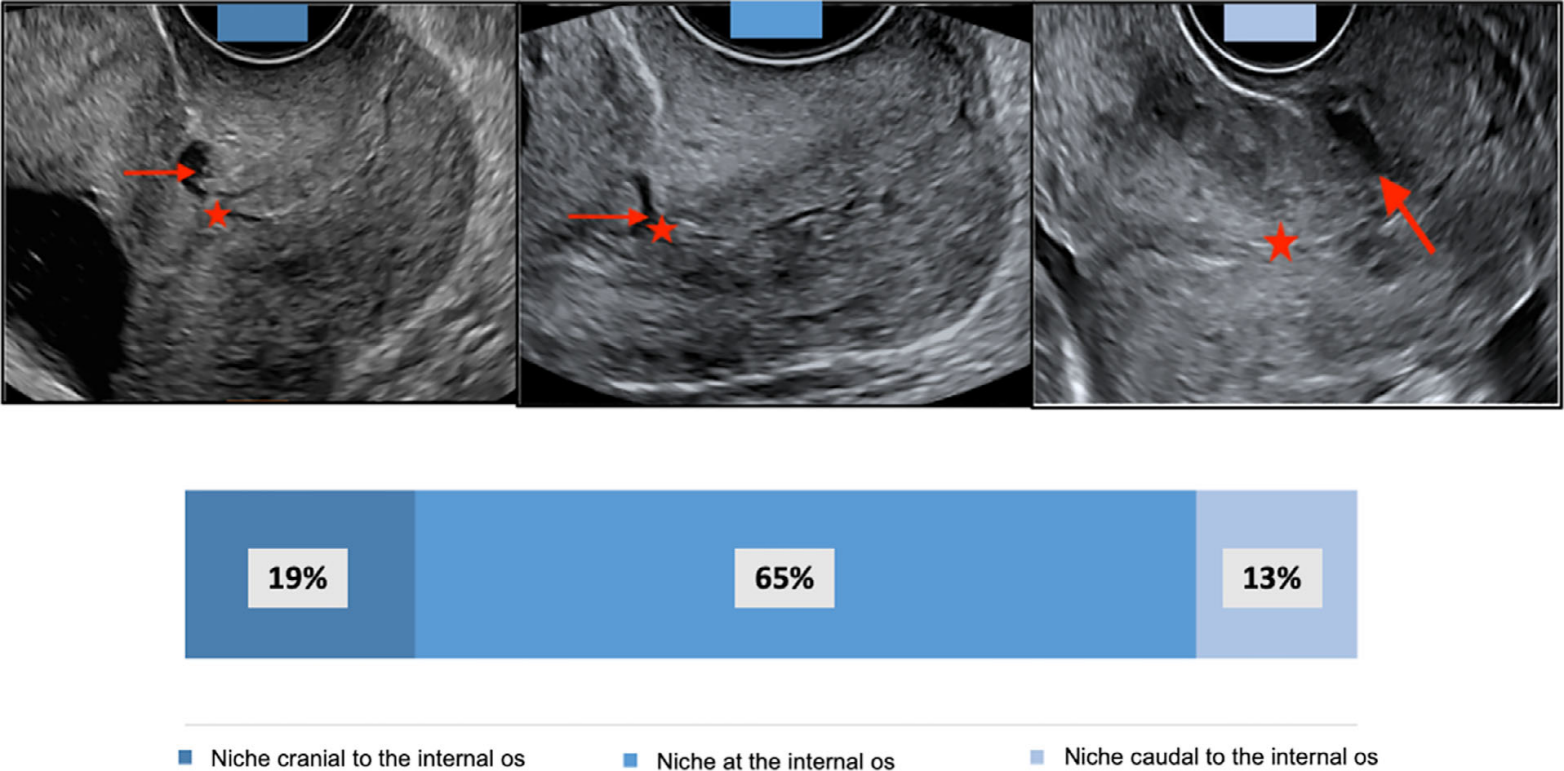
The sonographic position of CB scar niches relative to the level of the internal cervical os (red star: internal cervical os; red arrow: niche).

There was 100% agreement between the 2 operators regarding CB scar niche visualization on 3D TV US in all selected cases. The intraobserver level of agreement on niche classification in relation to the internal os was 100%. The interobserver kappa coefficient for assessment of niche position relative to the internal os was 0.98 (51/52, 95% confidence interval [CI] 0.97–0.99, *P* < .05) and there was disagreement in 1 case regarding niche classification. The sonographic appearance of niche position relative to the internal os revealed that 65% (34/52) cases had the niche located at the level of the internal cervical os. A total of 19% (10/52) and 13% (7/52) CB scars had the niche cranial and caudal to the internal cervical os, respectively (Figure 6).

**Figure 7:**
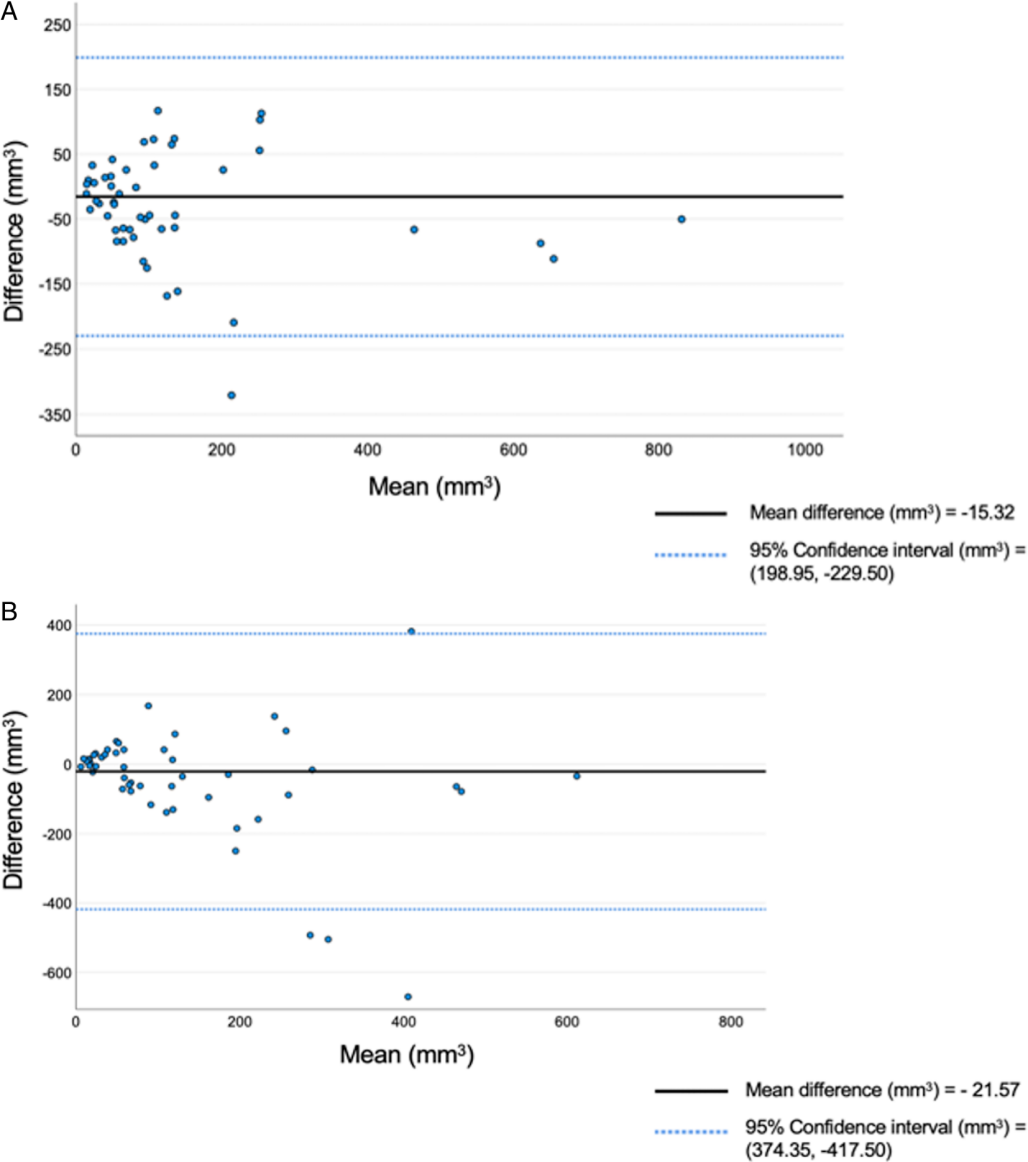
Bland–Altman plots of intra‐ (top) and inter‐ (bottom) observer reproducibility of niche volume.

The intraobserver reproducibility for niche volume was very good at ICC of 0.94 (95% CI 0.90–0.97; *P* < .001) and small mean difference of −15.32 mm^3^ (±109.32, 95% CI 198.95–229.50). Furthermore, the interobserver reproducibility for niche volume analysis demonstrated good level of agreement between operators, the ICC was 0.78 (95% CI 0.62–0.87; *P* < .001) and the mean difference was −21.57 mm^3^ (±202.01, 95% CI −417.50, 374.35). The corresponding Bland–Altman analysis is illustrated in Figure 7. Using a predefined cut‐off extrapolated from a comparable study conducted in nonpregnant population,^30^ 92% (48/52) of intraobserver and 88% (46/52) of interobserver differences measured ≤0.25 cm^3^.

Niche/cervix ratio mean difference (±SD) was −0.06 ± 0.62 (95% CI −0.68, 0.56; *P* < .001) for intraobserver reproducibility and −0.08 ± 1.14 (95% CI −1.22, 1.07; *P* < .001). The median of the niche volume distribution was 93.33 mm^3^ (range 10.67–1276.67 mm^3^), which relates to a cube with a side of 4.54 mm length. The 25th and 75th percentile were 51.75 mm^3^ (cube with a side of 3.72 mm) and 209.91 mm^3^ (cube with a side of 5.94 mm), respectively. The distribution of all niche measurements included in this study can be found in Table 2.

**Table 2:** Summary of the Ranges, Standard Deviations, and Percentiles for Niche Measurements in the Study

	Range	SD	25th centile	50th centile	75th centile
Niche length (mm)	2.50–22.00	±3.40	4.48	6.15	8.09
Niche depth (mm)	2.00–19.73	±3.05	4.31	5.73	7.54
Niche width (mm)	2.43–18.97	±3.25	4.66	6.28	7.78
Niche residual tissue thickness (mm)	1.97–10.47	±1.82	3.87	5.01	6.09
Niche adjacent tissue thickness (mm)	3.53–21.5	±3.30	7.20	9.71	12.22
Niche to internal os (mm)^a^	−22.23–7.83	±5.66	−4.62	0	0
Niche to external os (mm)^a^	14.17–43.8	±6.34	27.64	31.13	35.4
Cervical length (mm)^a^	26.27–39.97	±3.92	28.82	32.70	35.96
Niche volume (mm^3^)	10.67–1276.67	±211.49	51.75	93.33	209.91
Cervix volume (cm^3^)	11.85–55.05	±9.45	27.64	34.85	40.19
Niche/cervix ratio^b^	0.03–3.56	0.62	0.13	0.28	0.63

SD, standard deviation.

^a^ Shortest value recorded.

^b^ Niche volume/cervix volume × 100. Negative values assigned to niches below the level of the internal os.

## Discussion

In this prospective study, we have developed a reproducible protocol for 3D niche volume US assessment of CB scar niche characteristics in an ethnically diverse cohort of women during the first and second trimester of the subsequent pregnancy, at a median interval of 3 years from the previous term emergency CB. The scar niche was adequate for analysis by both operators in all included cases and there was high level of agreement for assessment of niche position relative to the internal os on 3D volumes. Our intra‐ and interobserver reproducibility study for niche volume and niche:cervix ratio demonstrated very good and good level of agreement, respectively, with narrow ranges. By using a standardized plane for volume acquisition, which included color Doppler assessment of uterine artery to aid identification of the level of the internal cervical os, we obtained highly reliable reconstruction planes.

To our knowledge there is no published data describing 3D niche evaluation, and in particular niche:cervix ratio volume assessment in pregnancy. A smaller study investigating the reproducibility of 3D US for the measurement of CB scar niche volume conducted in nonpregnant women (n = 20) reported ICC of 0.87 (95% CI 0.71–0.95) and ICC of 0.79 (95% CI 0.54–0.91) for intra‐ and interobserver agreement, respectively.^30^ Although the results are comparable to our study findings in pregnant women, the authors did not report on the assessment of niche position relative to the internal cervical os, nor on the relationship between niche and cervix volume. It has been suggested that in pregnant women, the presence of amniotic fluid can act as a contrast agent and therefore improve the visibility of the margins of the CB scar niche.^31^

Reproducibility studies on cervical volume alone using VOCAL or geometric formulae have demonstrated high levels of agreement in pregnant and nonpregnant women.^19^, ^20^, ^32^ A study that developed reference ranges for cervical volume using 3D US in pregnant women at low risk of sPTB concluded that there was no significant variation in cervical volume between 17 and 40 weeks' gestation.^21^ In our study, we demonstrated that the niche:cervix ratio can be assessed reliably in pregnancy. Although recent literature evidence has established that CB when the cervix is fully dilated increase the risk of recurrent LM and SPTB in future pregnancies,^3^, ^4^, ^5^, ^6^, ^7^ the pathophysiology of this association is poorly understood. Literature evidence demonstrates that the greater the cervical dilatation during the index CB, the higher the risk of subsequent sPTB, with the risk significantly increasing when the surgical procedure is performed in advanced labor at ≥8 cm cervical dilatation.^5^, ^33^ Unintended structural damage to the cervix at the time of CB, due to inadvertent incision of the cervix or cervical/uterine extensions, may lead to an incomplete recovery of the mechanical function of the cervix in subsequent pregnancies.^34^ In women with full dilatation CB, the position of the scar relative to the internal os on 2D TV US appears to be an important predictor of cervical length shortening and/or sPTB in subsequent pregnancies.^35^

Our study demonstrates that when volume acquisition is done in a well‐defined mid‐sagittal cervical starting plane, reconstruction of 2D measurements planes for niche contouring can be reliably achieved. Our observed mean differences for niche volume were small for both intra‐ and interobserver assessment. This technique is supported by previous studies that demonstrated good level of agreement between measurements originating from 3D volumes and those obtained during real time 2D scanning. Quality assessment studies of sonographic image acquisition suggested that reproducibility is greater when standardized image scoring system is used, compared to subjective assessment, both in 2D and 3D ultrasonography.^16^, ^26^, ^27^, ^28^, ^29^

Similarly, as described in our study design, we propose a standardized 3D volume acquisition technique that includes objective key anatomical landmarks with compulsory visualization in the plane of acquisition. In our study, both operators received structured training and practiced volume analysis beforehand. Likewise, other reproducibility studies on training sonography operators also showed high levels of agreement for predefined measurements.^24^

Another strength of our study is that we assessed the niche position relative to the cervix on 3D US volumes. Although the location of the internal os may be challenging to define on still images, this study demonstrates that, by employing a combination of methods described in the study protocol, standardized volume acquisition and dynamic assessment of the cervical planes on 3D volumes, the level of the scar niche in relation to the internal os can be reproducibly assessed. The intra and interobserver kappa coefficients for assessment of niche position in relation to the internal os were highly reproducible. These findings support our recently published study that reported the distance from the closest niche margin to the internal os was the most reproducible measurement on 2D TV US assessment.^16^ The added value of 3D assessment of CB scar is represented by quantification of niche volume and its rapport with cervical volume.

We acknowledge that this study did not include any cases of cervical shortening or funneling. In our clinical practice, we observed that CB scar niches are poorly visualized once funneling develops, due to tension forces that occur at the level of the niche. Preselection of good‐quality 3D US volumes may limit the generalizability of our findings to routine clinical settings, where image quality can vary significantly. In this study, we included all consecutive cases in which a niche was present, regardless of scar location, niche size or other characteristics, to reflect clinical practices. However, 2 cases were excluded due to inappropriate acquisition angles, which limited niche visualization and contouring in some reference planes.

Another potential limitation resides in the offline volume processing which differs from real‐time settings, where the absence of time constraints can affect reproducibility. The clinical relevance of 3D US scar niche volume requires further investigation. Incorporating artificial intelligence models and machine learning could help automate this process, potentially improving accuracy and reducing errors in real‐time clinical practice. We believe that the high reproducibility observed is attributed to the standardized 3D volume acquisition technique, which included objective anatomical landmarks, combined with the rigorous training the operators received. It is likely that the high level of experience among the operators contributed to the high reproducibility results.

In conclusion, this study demonstrates that 3D niche volume, niche volume relative to cervical volume, as well as position of the niche in relation to the internal os, can be reliably assessed in pregnancy. This is an important first step in determining whether 3D US features of CB scars may have a role in the development of algorithms to predict serious pregnancy complications such as cesarean scar pregnancy, abnormally invasive placenta, sPTB/LM or uterine rupture.

## Data Availability

The data that support the findings of this study are available on request from the corresponding author. The data are not publicly available due to privacy or ethical restrictions.
